# A Comparative Analysis of Acute Physiological and Perceptual Responses in Whole-Body and Ergometer-Based High-Intensity Interval Training Protocols

**DOI:** 10.3390/sports12060166

**Published:** 2024-06-14

**Authors:** Gustavo Z. Schaun, Rafael B. Orcy, Fabrício B. Del Vecchio

**Affiliations:** 1 Centre for Sport Science and University Sports, Department of Sport and Human Movement Science, University of Vienna, 1150 Vienna, Austria; 2 Physical Education School, Universidade Federal de Pelotas, Pelotas 96010-610, RS, Brazil; rafaelorcy@gmail.com (R.B.O.); fabricioboscolo@ufpel.edu.br (F.B.D.V.)

**Keywords:** exercise, aerobic training, intermittent exercise, physical exertion, muscle injury

## Abstract

The primary aim of the present investigation was to compare the acute physiological and perceptual responses between two modes of interval training using a randomized crossover design. More specifically, eleven young adult participants (23 ± 4 years, 77 ± 13 kg, 178 ± 7 cm) performed two protocols: one composed of whole-body calisthenics exercises and another on a cycle ergometer. Both protocols encompassed eight 20 s bouts at intensities equivalent to all-out (HIIT-WB) and 170% of the maximal power output (HIIT-C), respectively, interspersed with 10 s of passive rest. The peak and average heart rate, the rating of perceived effort, and blood lactate, creatine kinase, and lactate dehydrogenase concentrations were measured. Aside from blood lactate (HIIT-WB = 9.4 ± 1.8 mmo/L; HIIT-C = 12.5 ± 2.5 mmol/L, *p* < 0.05) and the rating of perceived exertion (HIIT-WB = 8.8 ± 0.9; HIIT-C = 9.6 ± 0.5, *p* < 0.05), physiological responses did not significantly differ between protocols (all *p* > 0.05), with high average heart rate values (HIIT-WB = 86 ± 6% HRmax; HIIT-C = 87 ± 4% HRmax) and a low magnitude of muscle damage, as inferred by CK and LDH concentrations (HIIT-WB = 205.9 ± 56.3 and 203.5 ± 72.4 U/L; HIIT-C = 234.5 ± 77.1 and 155.1 ± 65.3 U/L), respectively. It can be concluded that both protocols elicit vigorous heart rate responses and a low magnitude of muscle damage and, therefore, appear as viable alternatives to improve aerobic fitness. The inclusion of a whole-body HIIT protocol may be an interesting alternative for training prescription in relation to more common interval training protocols.

## 1. Introduction

High-intensity interval training (HIIT) involves short bursts of intense exercise interspersed with periods of lower intensity or rest [1]. This training method has been extensively studied and has led to the development of numerous protocols due to its effectiveness and time efficiency [2]. Recently, low-volume HIIT protocols have gained popularity because they deliver significant fitness benefits in a relatively short amount of time [3,4,5].

Traditionally, HIIT is performed using equipment such as treadmills or cycle ergometers. However, these tools are not always accessible to everyone. To make HIIT more feasible for the general population, alternative modes of training that require minimal equipment are needed. Bodyweight exercises, which can be executed in diverse environments, from parks to constrained spaces like garages or living rooms, present a cost-effective and practical alternative to traditional ergometer-based HIIT [6]. This approach aligns with current trends; “Body weight training”, “Outdoor Activities”, and “High-intensity interval training”, for example, have been frequently ranked among the top fitness trends in the industry [7].

Despite the growing interest in bodyweight HIIT, there is limited information on the acute physiological and perceptual responses to this exercise mode. Previous research has shown that bodyweight HIIT can elicit high heart rate responses. For example, McRae et al. [8] reported heart rates above 80% of maximum HR during bodyweight HIIT in young women. Additionally, our laboratory demonstrated that 16 weeks of bodyweight HIIT can improve maximal oxygen uptake (VO_2_max) to a similar extent as volume-matched treadmill HIIT [6]. However, direct comparisons of the acute responses between bodyweight and ergometer-based HIIT protocols remain scarce. Understanding these responses, including heart rate (HR), rating of perceived exertion (RPE), blood lactate concentration (BLa), and muscle damage markers, is crucial for optimizing training programming.

Therefore, the purpose of this study was to compare the acute physiological and perceptual responses of two HIIT protocols known for improving cardiorespiratory fitness and matched for total exercise duration: one using bodyweight exercises and the other performed on a cycle ergometer. This comparison provides valuable insights for both practitioners and fitness enthusiasts, helping them choose appropriate HIIT protocols based on equipment availability and individual preferences. We hypothesized that, while heart rate responses and blood lactate levels would be similar between the two protocols, RPE and muscle damage markers would differ between them.

## 2. Materials and Methods

### 2.1. Experimental Design

**Figure 1:**
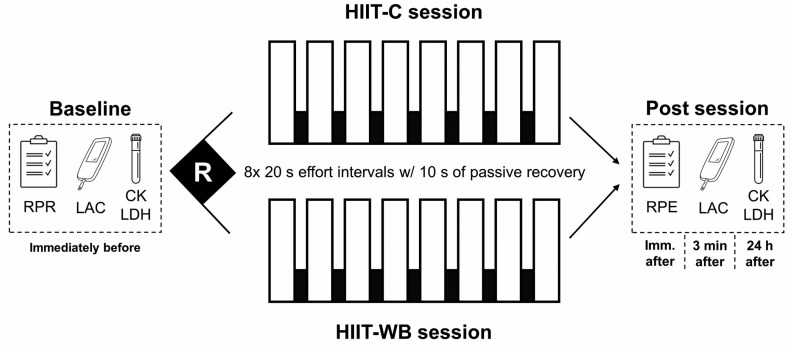
Experimental design. HIIT-C = ergometer-based high-intensity interval training; HIIT-WB = whole-body high-intensity interval training, RPE = rating of perceived effort; RPR = rating of perceived recovery; LAC = blood lactate concentration; CK = creatine kinase; LDH = lactate dehydrogenase.

The investigation was conducted in three separate sessions (Figure 1). During the first visit, participants warmed up on a cycle ergometer (Biotec2100^®^, Cefise, Nova Odessa, Brazil) for 10 min at 60% of their estimated HRmax [9] and rested for five minutes before performing an incremental cycle ergometer test. The test began with a load corresponding to 0.60 kp with increments of 0.25 kp each minute. The test was terminated when the individual volitionally stopped exercise owing to fatigue or the investigator determined that the subject could not maintain a pedal rate greater than 50 rev/min for 10 consecutive seconds. The last stage completed by each participant was used to estimate their V̇O_2_max using Storer’s equation [10,11]. Considering that oxygen uptake was not measured directly, a plateau in oxygen consumption or a respiratory exchange ratio cut-off value could not be used to confirm the attainment of a maximal test. Nevertheless, all participants investigated achieved peak heart rate measures within ± 10 beats of their estimated HRmax and reported an RPE between 9 and 10 at the end of the test. All participants were asked to avoid vigorous exercise for 48 h, and caffeine, alcohol, or any stimulant 24 h before the sessions. They were also asked to maintain their normal nutritional intake throughout the experiment and to eat their last meal ~3 h before the tests. After the first session, a randomized crossover design was adopted to determine the order of the two experimental sessions, two weeks apart from each other. More specifically, the order of the sessions was determined by simple randomization using a custom-built random number generator spreadsheet in Microsoft Excel^®^. Compliance with the aforementioned recommendations was confirmed by the participants immediately before each session.

### 2.2. Participants

Eleven recreationally active young male adults signed an informed consent form and were enrolled in the study (23.3 ± 3.9 years, 76.9 ± 12.9 kg, 177.8 ± 7.4 cm, VO_2_max = 52.6 ± 5.9 mL·kg^−1^·min^−1^). More specifically, participants were physically active and engaged regularly (≥2x/wk) in different forms of physical exercise but were not considered athletes. Those who presented muscle or joint pain in the last three months, cardiometabolic diseases, or were using medications that could interfere with the outcomes of interest were not included. Compliance with the aforementioned criteria was confirmed by a detailed anamnesis. Moreover, the research project was previously approved by the institution’s Research Ethics Committee in accordance with the Helsinki Declaration and written informed consent was received from all participants before participation.

### 2.3. Procedures

#### 2.3.1. Experimental Sessions

In both the second and third sessions, participants warmed up for five minutes at 60% of their HRmax and rested for five minutes before performing the protocol assigned for that visit. Each protocol was composed of eight 20 s effort bouts interspersed with 10 s passive recovery intervals. Ergometer-based HIIT (HIIT-C) was performed on the same cycle ergometer as the incremental test and effort bouts were performed at 170% Pmax and ~90 rpm [12]. Whole-body high-intensity interval training (HIIT-WB), on the other hand, was performed using whole-body calisthenics exercises, i.e., burpees, mountain climbers, jumping jacks, and squat and thrusts with 3.1 kg dumbbells, at an all-out intensity [6]. More specifically, during HIIT-WB participants executed only one exercise per effort bout in a pre-defined sequence (i.e., burpees, mountain climbers, jumping jacks, and squats and thrusts) and repeated this sequence twice for a total of eight bouts. Given the potential variability in physiological responses induced by different whole-body exercises, we specifically selected these exercises for their demonstrated impact on both aerobic and anaerobic systems [6,8]. During both HIIT-C and HIIT-WB, participants received strong verbal encouragement to ensure that a maximal effort was given.

#### 2.3.2. Outcomes of Interest

To investigate the acute responses of these protocols, peak (HRpeak) and average (HRavg) HR were measured using a heart rate monitor (RS800CX, Polar Electro, Kempele, Finland) and are expressed relative to the HRmax and HR reserve. Moreover, approximately 5 μL of blood was sampled from the participant’s earlobe before and three minutes after the end of the experimental protocols for BLa determination using a portable lactometer (LactatePlus, NOVA Biomedical™, Waltham, MA, USA). An additional 5 mL blood sample was collected from the antecubital vein before and 24 h after each protocol for CK and LDH determination. Blood was stored in heparinized tubes, which were centrifuged and stored at −20 °C until further analysis using commercial kits for CK (bio-LIFE^®^, Belo Horizonte, Brazil) and LDH (Diagnostic Labtest SA, Lagoa Santa, Brazil). The total concentration of the two enzymes was measured using a spectrophotometer (San Jose, CA, USA SpectraMax^®^), using a colorimetric UV method at a wavelength of 340 nm according to the manufacturers’ recommendations. Finally, to ensure that participants were adequately recovered before each experimental session, their recovery status was assessed using the Perceived Recovery Status Scale, as previously recommended [13]. During their first visit, all participants received instructions on how to use the scale. Before each experimental protocol, the scale was presented to the participants while they were resting comfortably in a quiet environment. In turn, RPE was measured after HIIT-C and HIIT-WB using the OMNI Picture System of Perceived Exertion, which was presented to participants following previous recommendations [14]. All participants were already previously familiarized with the RPE scale.

### 2.4. Statistical Analysis

All variables were tested for normality and homogeneity of variances with the Shapiro–Wilk and Levene tests, respectively, and are presented as means and standard deviations (±SD). HR, RPE, and the rating of perceived recovery were compared between protocols using t-tests for dependent variables, whereas BLa, CK, and LDH were compared using two-way repeated measures ANOVA (2 groups × 2 time points) and Bonferroni’s post hoc. In the case of significant group × time point interactions, simple main effect analyses were used to test the main factors. Correlations between CK and LDH and also between both enzymes’ concentrations and HRpeak, HRavg, and RPE were performed using Pearson’s product moment test. An ɑ = 0.05 was adopted for all analyses and within-subject effect sizes (ES) were calculated using Cohen’s d_rm_ according to previous recommendations [15] and considered as small (0.2), medium (0.5), and large (0.8).

## 3. Results

As presented in Table 1, no difference in the rating of perceived recovery was observed between HIIT-C and HIIT-WB (*p* = 0.14; ES = 0.44), demonstrating that participants began the experimental sessions at similar conditions. Moreover, both protocols resulted in similar HRpeak (*p* = 0.57; ES = 0.28) and HRavg (*p* = 0.73; ES = 0.19) responses, whereas RPE was higher after the HIIT-C protocol (*p* = 0.02; ES = 1.21).

**Figure 2:**
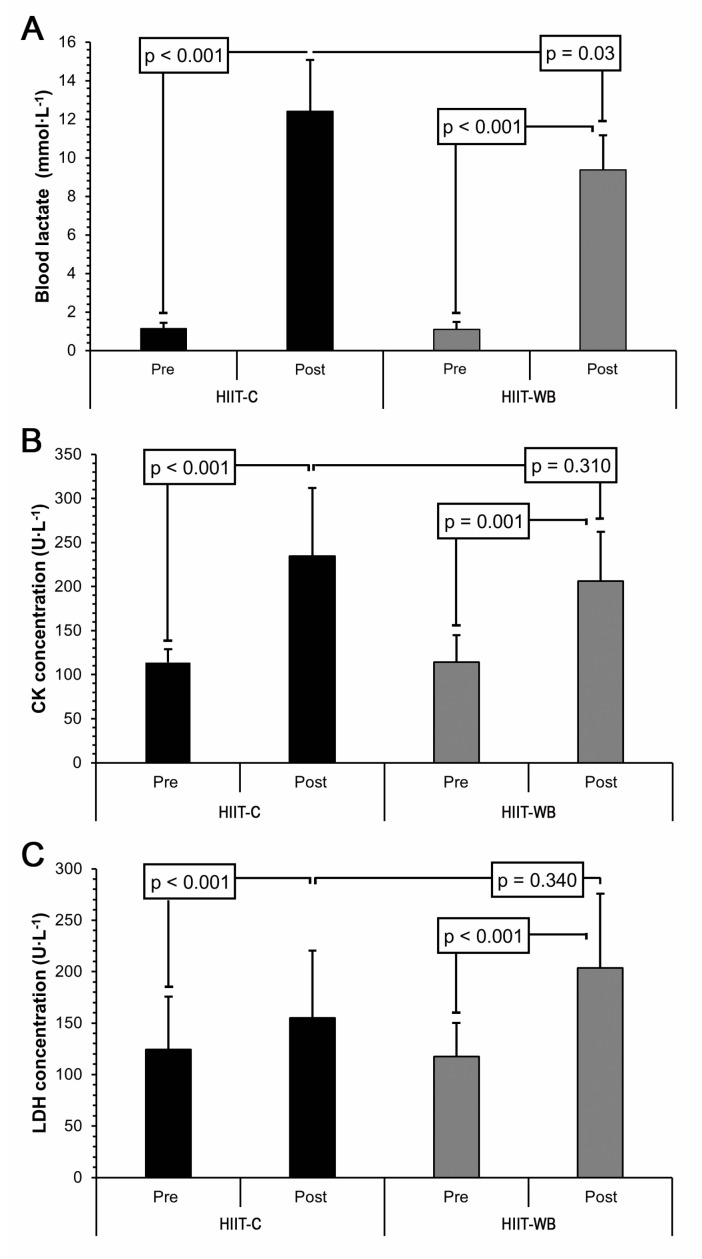
Blood lactate (**A**), creatine kinase (**B**), and lactate dehydrogenase (**C**) results pre- and 3 min (BLa) or 24 h (CK and LDH) post session according to the protocol performed in healthy young male adults (n = 11). HIIT-C ergometer-based high-intensity interval training; HIIT-WB whole-body high-intensity interval training.

A significant group × time point interaction (*p* = 0.005) was observed for BLa concentration, as shown in Figure 2A. Simple main effects analysis showed that BLa concentrations increased after both HIIT-C and HIIT-WB (both *p* < 0.001); however, post-exercise values differed according to the protocol performed (*p* = 0.03; ES = 1.21). More specifically, a higher BLa concentration was observed after HIIT-C (12.5 ± 2.5 mmol/L) compared to HIIT-WB (9.4 ± 1.8 mmol/L).

CK and LDH results are presented in Figure 2B,C, respectively. When compared to the baseline, CK increased 24 h after both HIIT-C and HIIT-WB (both *p* ≤ 0.001), but no difference was found between protocols (*p* = 0.31; ES = 0.56). A similar pattern was also found for LDH, i.e., significantly higher values were found 24 h after both protocols (both *p* < 0.001), which were not significantly different from each other (*p* = 0.34; ES = 0.74). As for the correlations, LDH and CK values were found to be significantly associated (r = 0.64; *p* = 0.02). Additionally, CK was also positively correlated with HRpeak in HIIT-C (r = 0.57; *p* = 0.04) and HIIT-WB (r = 0.57; *p* = 0.05). No correlations were observed for RPE (r = 0.38; *p* = 0.22) or HRavg (r = 0.39; *p* = 0.20).

## 4. Discussion

The main finding of the present investigation was that both HIIT-C and HIIT-WB resulted in similar cardiovascular responses, although the effort was perceived differently between the two training modes. In addition, CK and LDH results were not different between protocols and their values suggest a low magnitude of muscle damage.

Heart rate responses averaged approximately 86% HRmax and 80% HRR during both HIIT-C and HIIT-WB. According to established guidelines, these results indicate that the aforementioned protocols can be classified as vigorous and, therefore, appear as viable alternatives to be implemented in exercise programs aimed at improving cardiorespiratory fitness and reducing cardiometabolic risk [16]. Previous investigations showed that a HIIT-WB protocol composed of four 30 s all-out bouts of burpees and 4 min of active recovery elicited ~85% %HRmax [17], while McRae et al. [8] observed responses between 83 and 90% HRmax when all eight bouts of our HIIT-WB protocol were performed using just one exercise. As such, by maintaining the work-to-rest ratio, it seems that the HIIT-WB exercise sequence can be modified without a decrease in cardiac demand and intensity. Consequently, as it does not require expensive equipment and can be performed in different settings, this HIIT-WB protocol may serve as a useful time-efficient alternative to overcome both the lack of time barrier to exercise engagement [4,6] and the lack of resources. HIIT-WB may also potentially be used within the framework of “exercise snacks”, which has recently been introduced in the training literature [5,18]. This approach may not only help in improving fitness, but also serve as a practical tool for breaking up sedentary behavior, which independently impacts cardiometabolic risk [5].

Nevertheless, even though the protocols elicited similar HR responses, participants perceived HIIT-C as more demanding when compared to HIIT-WB. This is in accordance with the only study we are aware of that compared RPE between ergometer-based and calisthenics protocols [17]. As mentioned previously, after four 30 s all-out bouts with 4 min of active rest, RPE was significantly lower when the protocol was performed using burpees compared to cycling (14.5 vs. 17, respectively). In this regard, cycling generally leads to larger peripheral fatigue compared to running, partially because the fractional duration of external force application during cycling results in greater blood flow restriction to the lower limbs [19], which may enhance peripheral fatigue [20]. Accordingly, it may be that during whole-body exercises, such as those in HIIT-WB, the fractional duration of external force application by the same muscle groups (e.g., knee extensors) was reduced and could have potentially resulted in lower fatigue and, consequently, lower RPE. Alternatively, it could also be argued that, during whole-body exercises, the recruitment of different muscle groups could result in competition and reduced blood flow distribution when compared to muscles working in isolation [21]; thus, this remains to be investigated.

Despite identical work-to-rest ratios in both protocols, the significantly higher BLa post-HIIT-C may indicate heightened metabolic stress and enhanced anaerobic glycolysis compared to HIIT-WB. In this regard, the inclusion of jumping jacks in the HIIT-WB protocol may have led to a decrease in the protocol’s overall intensity since it was shown to result in significantly lower RPE and fatigue of the upper and lower limbs compared to burpees, mountain climbers, and squat and thrusts [8]. This may be at least partially explained by the smaller muscle mass demand or engagement during jumping jacks [22]. Additionally, the muscle groups exercised during HIIT-C were virtually the same across all effort bouts, while during HIIT-WB the movement pattern changed significantly throughout the different exercises performed. This means that muscle groups recruited during HIIT-WB may have differed between bouts, possibly allowing greater recovery until the same muscle group was recruited again [22]. In addition to the differences in BLa concentration, these aspects could also serve to explain the differences in perceived effort that were observed between the two protocols, despite the absence of difference in the heart rate responses. That being said, both HIIT-C and HIIT-WB results corroborate the notion that the protocols were indeed performed at high intensities by the participants included.

To the best of the authors’ knowledge, no study to date has investigated the response of indirect muscle damage markers to HIIT-WB. Since it presents marked eccentric and concentric phases and is composed of high-impact exercises instead of strictly cyclic activities, differences between the investigated protocols were expected. Nevertheless, contrary to our initial hypothesis, no differences were observed between HIIT-C and HIIT-WB. While our findings indicated low muscle damage, contrasting results have been observed in studies involving plyometric or sprint-based protocols, which reported higher elevations in muscle damage markers. These discrepancies could be attributed to the different mechanical demands imposed by these types of exercises. Previous studies that investigated repeated sprint training protocols found marked elevations in CK concentration [23,24,25]. As an example, a considerable increase in CK (~776 U/L) was observed 24 h after fifteen all-out 30 m sprints [25]. When compared to our results, sprints are known to generate great mechanical stress on lower limb muscles as participants try to decelerate the body [23,26]. This deceleration component (i.e., eccentric) was not present during HIIT-C and possibly at a lower magnitude during HIIT-WB. This notion is partially supported by Twist and Eston [27], who observed just a minor increase in CK (151 U/L vs. 239 U/L) 24 h after 10 sets of 10 maximal vertical jumps.

Furthermore, we also observed an increase in LDH concentration above the baseline in both HIIT-C and HIIT-WB, although it remained within the enzyme’s resting value for adults. These findings agree with the previous notion that exercise intensity exerts only a minor effect on muscle damage when exercise volume is not high enough [28] and appears to hold even when interval training is performed at supramaximal and all-out intensities, as shown in the present investigation. More recently, 12 min (15:15 s) of HIIT at 100% VO_2_max was shown to induce likely small increases in indirect muscle damage markers such as CK [29]. Taken together, the small increases observed in CK and LDH suggest a low magnitude of muscle damage in both protocols tested. It should be noted, however, that we were unable to assess muscle soreness, muscle function, or inflammation markers and caution is thus needed when interpreting these findings. For example, a low magnitude of muscle damage based solely on blood muscle damage markers does not necessarily infer that muscle function is completely restored. In addition, although participants were requested to perform HIIT-WB exercises at an all-out intensity and were strongly verbally encouraged to do so, there is currently no objective parameter to control all-out training stimulus.

As for the correlations, CK and LDH were moderately correlated (r = 0.64), and although both are cytoplasmic molecules that are released in response to sarcoplasmic membrane damage [30], their appearance in the bloodstream was not similar. When indirect muscle damage markers were correlated to variables related to the protocols’ intensity, only CK showed significant results. More specifically, moderate correlations with HRpeak in HIIT-C and HIIT-WB protocols were found, which were able to explain approximately 40% of CK variance in each protocol. These results are in line with the notion that intensity is relevant for inducing muscle damage, but other variables such as total duration and contraction type are as important to determine such a response [31]. Curiously, no correlations were established between both enzyme concentrations and RPE, which are commonly accepted as general markers for internal load or intensity, supporting the assumption that the time-course of these outcomes is different.

The present study is not without limitations. First, although the exercise duration between the two training sessions was similar, the intensity between the two protocols was not matched given the nature of the HIIT-WB session employed and the inherent difficulty of measuring the physical work completed during this type of training. We also acknowledge the absence of an appropriate a priori sample size estimation and the fact that the sample size in our study was rather small. However, the use of a within-subject design helps mitigate this issue by reducing the variability due to interindividual differences that would have been present with a between-subjects design. Lastly, it should be noted that only young men were included and, therefore, the results of this study may not directly translate to the general population.

## 5. Conclusions

In summary, both protocols investigated elicited vigorous HR responses and, therefore, appear as viable alternatives to be implemented in exercise interventions aimed at improving aerobic fitness and reducing cardiometabolic risk. In this regard, low-volume HIIT-WB protocols represent an emerging alternative for professionals and practitioners that simultaneously addresses issues such as lack of time, space, resources, and security as it can be executed in diverse environments, including at home. Being perceived as less strenuous, HIIT-WB protocols may also favor adherence to this exercise mode when compared to more traditional HIIT protocols, although this should be confirmed in larger studies.

## Figures and Tables

**Table 1:** Heart rate and perceptual responses according to the protocol performed (n = 11).

	HIIT-C		HIIT-WB		
	Mean	SD	Mean	SD	*p* Value
HR_peak_ (bpm)	184.3	±7.8	182.1	±7.2	0.57
(%HRmax)	95.8	±4.1	94.8	±3.7	0.63
(%HRR)	93.9	±5.9	92.9	±6.2	0.63
HR_avg_ (bpm)	167.6	±8.5	165.6	±10.5	0.73
(%HRmax)	87.1	±4.3	86.1	±5.6	0.72
(%HRR)	80.9	±5.9	79.8	±9.6	0.72
RPR	6.8	±1.5	7.5	±1.5	0.14
RPE	9.6	±0.5	8.8	±0.9	0.02 *

HIIT-C ergometer-based high-intensity interval training; HIIT-WB whole-body high-intensity interval training; RPR rating of perceived readiness; RPE rating of perceived effort; HR_peak_ peak heart rate; HR_avg_ average heart rate; * = significantly different from HIIT-WB (*p* = 0.01). Note: HR responses are presented as beats per minute, percentage of the maximal heart rate (%HRmax), and percentage of the heart rate reserve (%HRR), respectively.

## Data Availability

The data supporting the conclusions of this manuscript will be made available upon reasonable request to the authors due to privacy and ethical restrictions.
